# Supplementary material for: Molecular pixelation of the CAR T cell surface proteome
Source: bioRxiv. 2026 Feb 2:2026.01.30.702970. Preprint. [Version 1] doi: 10.64898/2026.01.30.702970 (PMC12889555; doi:10.64898/2026.01.30.702970)
Supplement: Supplement 2 [file NIHPP2026.01.30.702970v1-supplement-2.pdf]

bioRxiv preprint doi: https://doi.org/10.64898/2026.01.30.702970; this version posted February 2, 2026. The copyright holder for this preprint (which was not certified by peer review) is the author/funder, who has granted bioRxiv a license to display the preprint in perpetuity. It is made available under aCC-BY 4.0 International license.

Supporting Figures

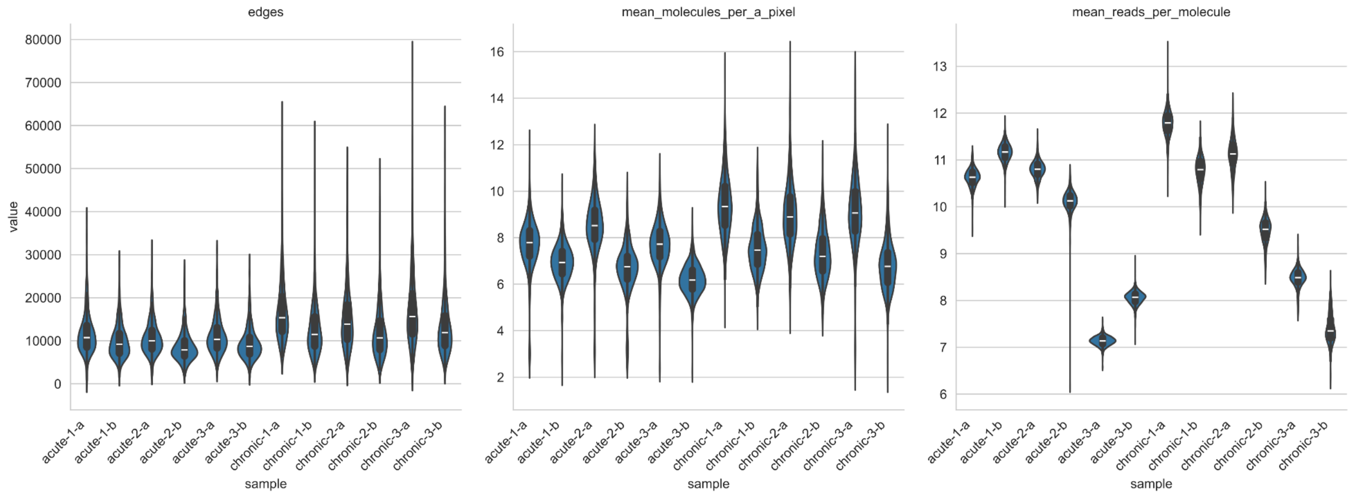

Supporting Figure 1. Quality control. A) Edges per cell, B) Mean molecules per pixel, C) Mean reads per molecule,

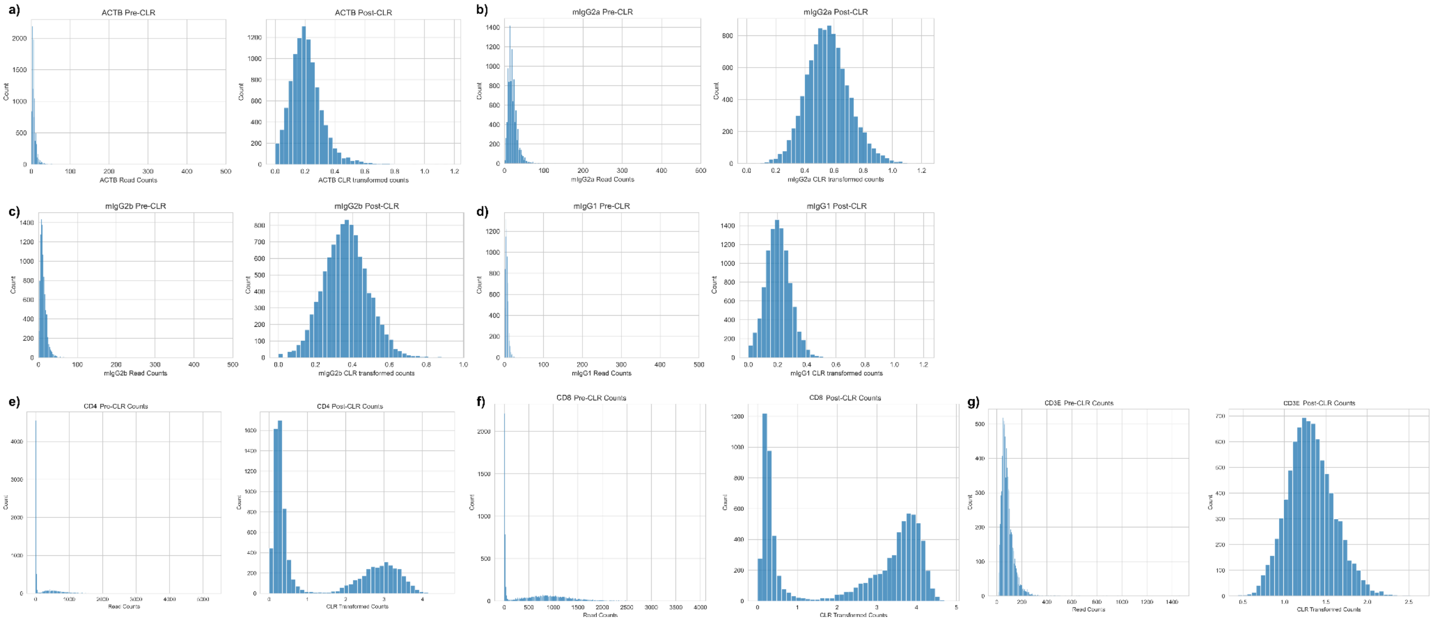

Supporting Figure 2. Normalization and comparison to controls. Intracellular control ACTB (a) and other controls mIG2a (b), mIG2b (c), and mIG1 (d) have low expression. Markers for CAR T cells CD4 (e) and CD8 (f) have bimodal populations representing two populations of these T cells, and the more ubiquitously expressed CD3E (g) is unimodal and highly expressed compared to the controls.

bioRxiv preprint doi: https://doi.org/10.64898/2026.01.30.702970; this version posted February 2, 2026. The copyright holder for this preprint (which was not certified by peer review) is the author/funder, who has granted bioRxiv a license to display the preprint in perpetuity. It is made available under aCC-BY 4.0 International license.

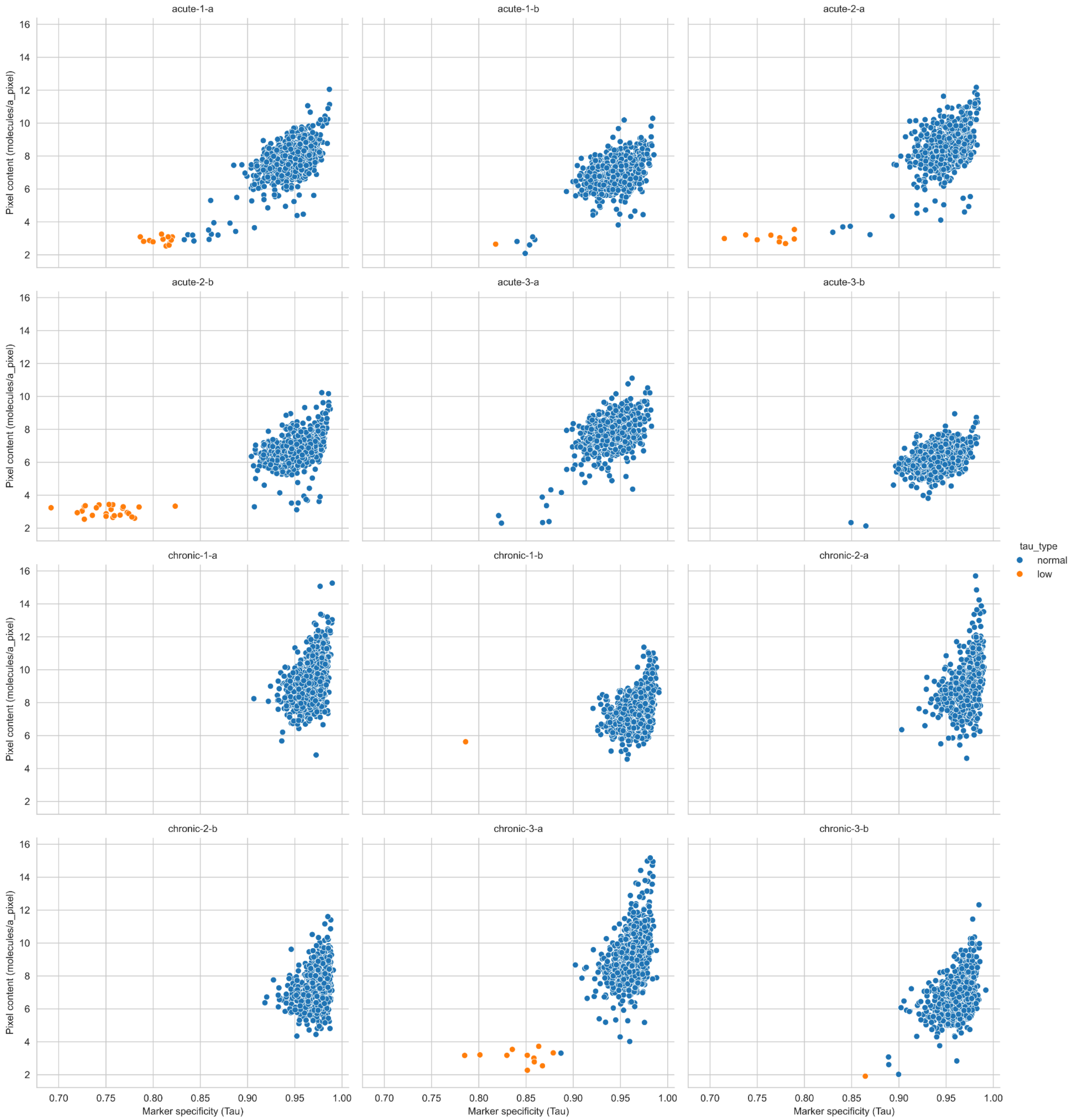

Supplementary Figure 3. The tau metric for marker specificity is used to identify aggregates (orange) with low specificity that were filtered from the cells that were used for the analysis (blue) with higher marker specificity. Individual plots represent individual samples of acute stimulation (top two rows), and chronic stimulation (bottom two rows).

bioRxiv preprint doi: https://doi.org/10.64898/2026.01.30.702970; this version posted February 2, 2026. The copyright holder for this preprint (which was not certified by peer review) is the author/funder, who has granted bioRxiv a license to display the preprint in perpetuity. It is made available under aCC-BY 4.0 International license.

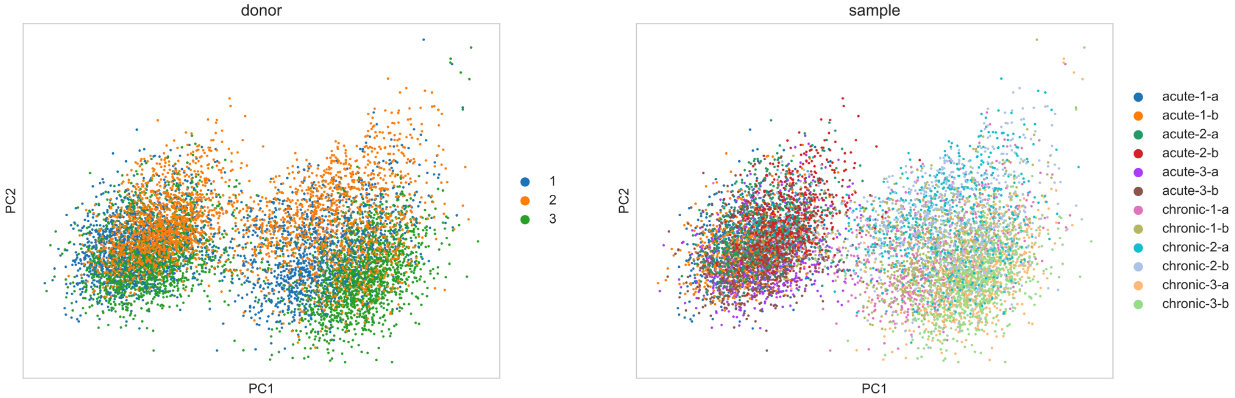

Supplementary Figure 4. Dimensionality reduction plots using PCA showing the distribution of donors and samples.

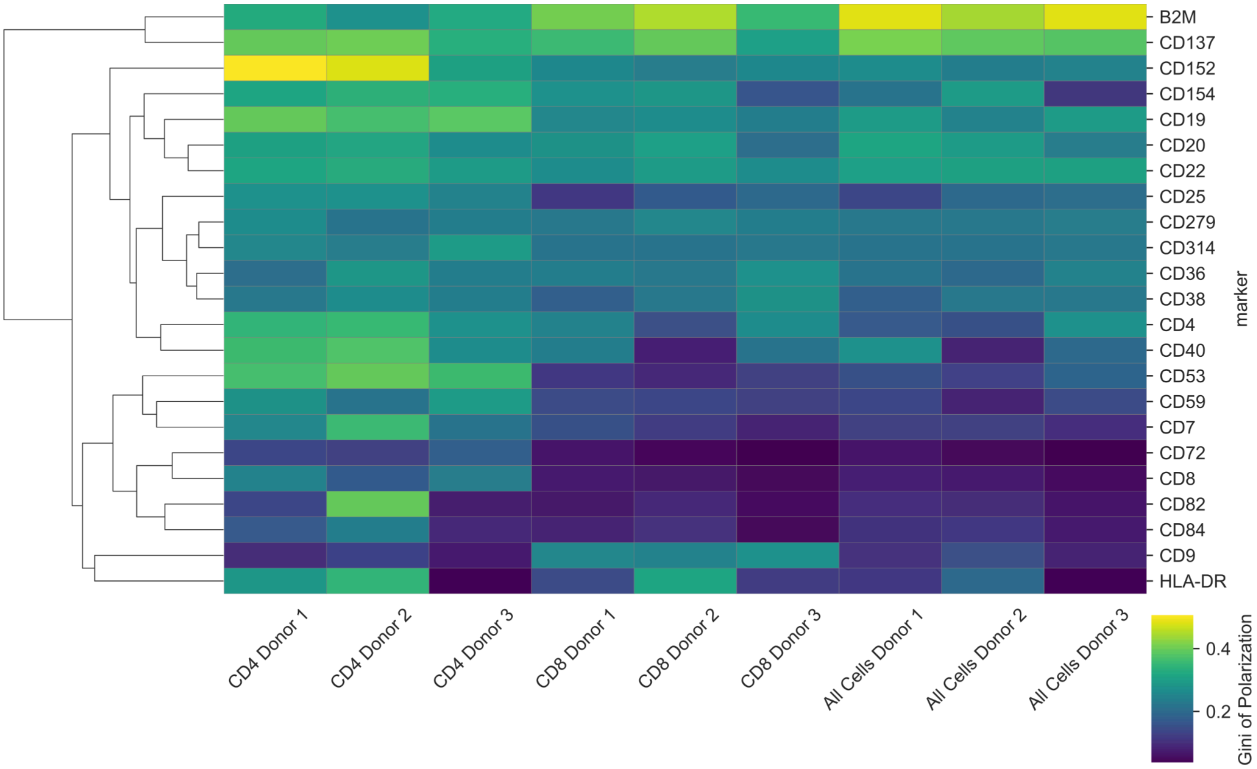

Supplementary Figure 5. Clustermap of the single-cell variability (Gini index) of polarization for markers with significant changes in the variability of polarization for all CAR T cells in the study. This metric is relatively stable across the three donors.
